# Adipogenesis of adipose-derived stem cells may be regulated via the cytoskeleton at physiological oxygen levels *in vitro*

**DOI:** 10.1186/scrt230

**Published:** 2013-07-09

**Authors:** Zachary A Schiller, Nathan R Schiele, James K Sims, Kyongbum Lee, Catherine K Kuo

**Affiliations:** 1 Department of Biomedical Engineering, Tufts University, Medford, MA, USA; 2 Department of Chemical and Biological Engineering, Tufts University, Medford, MA, USA; 3 Sackler School of Graduate Biomedical Sciences, Tufts University School of Medicine, Boston, MA, USA; 4 Science and Technology Center, Biomedical Engineering, Tufts University, 4 Colby St., Medford, MA, USA

**Keywords:** Adipogenesis, Cytoskeleton, Oxygen tension, Adipose-derived stem cells

## Abstract

**Introduction:**

Obesity, which is excessive expansion of white adipose tissue, is a major risk factor for several serious health issues, including diabetes, cardiovascular disease and cancer. Efforts to combat obesity and related diseases require understanding the basic biology of adipogenesis. However, *in vitro* studies do not result in lipid composition and morphology that are typically seen *in vivo*, likely because the *in vitro* conditions are not truly representative of *in vivo* adipose tissue formation. *In vitro*, low oxygen tension and cytoskeletal tension have been shown to independently regulate adipogenesis, but *in vivo*, these two factors simultaneously influence differentiation.

**Methods:**

The purpose of our study was to examine the influence of physiological oxygen tension on cytoskeletal tension-mediated adipogenesis. Adipose-derived stem cells (ASCs) were differentiated under both ambient (20%) and physiological (5%) oxygen conditions and treated with cytoskeletal inhibitors, cytochalasin D or blebbistatin. Adipogenesis was assessed on the basis of gene expression and adipocyte metabolic function.

**Results:**

Adipose tissue metabolic markers (glycerol-3-phosphate dehydrogenase (GPDH) and triglycerides) were significantly down-regulated by physiological oxygen levels. Reducing cytoskeletal tension through the use of chemical inhibitors, either cytochalasin D or blebbistatin, resulted in an up-regulation of adipogenic gene expression (peroxisome proliferator-activated receptor γ (PPARγ), lipoprotein lipase (LPL) and fatty acid binding protein 4 (FABP4)) and metabolic markers, regardless of oxygen levels. Cytochalasin D and blebbistatin treatment altered cytoskeletal organization and associated tension via different mechanisms; however, both conditions had similar effects on adipogenesis, suggesting that physiological oxygen-mediated regulation of adipogenesis in ASCs is modulated, in part, by cytoskeletal tension.

**Conclusions:**

These results demonstrated that interactions between the cytoskeleton and oxygen tension influence adipogenic differentiation of ASCs.

## Introduction

Obesity is a critical health crisis facing our society. As of 2008, more than 39% of U.S. adults over the age of 20 were obese [1]. This population has a higher risk for several diseases, including type 2 diabetes, cardiovascular disease and various cancers [2], resulting in medical costs of $147 billion annually [3]. Efforts to combat obesity and related diseases require an understanding of the basic biology of adipogenesis. While gene knockout models have revealed important insights into adipogenesis [4,5], *in vitro* experiments have been useful for elucidating the effects of individual factors, such as oxygen tension and tissue stiffness on adipogenic differentiation [6,7]. What is known about factors that regulate adipogenesis is based largely on differentiation studies with preadipocyte cell lines [8], mesenchymal stem cell lines [9] and primary mesenchymal stem cells (MSCs) [10]. Unfortunately, current *in vitro* models used for studying white adipose tissue expansion do not yet appear to completely replicate the *in vivo* adipogenesis process, indicating that additional factors involved in differentiation still need to be addressed.

During adipogenesis *in vitro,* 3T3-F442A mouse preadipocytes on tissue culture plastic undergo a morphological change, from fibroblastic to spherical, that appears to be critical for differentiation [8]. *In vivo*, preadipocytes appear spindle-shaped, while mature adipocytes grow in size and develop an oval or rounded morphology as lipid droplets accumulate intracellularly [11]. *In vitro* evidence suggest that this shape change occurs early in the differentiation process and prior to the up-regulation of many adipocyte specific genes as well as independently of triglyceride accumulation [12], though the cause and mechanism for the morphological shift from fibroblastic to spherical *in vivo* have yet to be determined. These morphological changes are accompanied by cytoskeletal changes, including decreased actin synthesis [8] and reorganization [13]. Altered actin organization may influence cytoskeletal tension, which has been shown to regulate adipogenesis in MSCs *in vitro*[14,15]. Though cytoskeletal changes appear to be critical to the differentiation process, the detailed mechanisms driving the morphological shift are not yet understood.

Environmental cues, such as oxygen tension, also factor into the regulation of adipogenic differentiation. Adipogenesis *in vitro* is typically performed in ambient air at 20% O_2_*.* In contrast, physiological O_2_ levels in adult adipose tissue from lean human patients range from 5.2 to 9.6%, while adipose tissue from obese human patients is even lower with O_2_ levels in the range of 3.8 to 8.2% [16]. These ranges coincide with reports that adipose tissue from lean mice has an average O_2_ level of 6.3%, while tissue from genetically modified obese mice average 2.0% [17]. Interestingly, published *in vitro* studies on adipogenesis as a function of oxygen tension have suggested that more physiologically relevant oxygen levels can inhibit adipogenesis [6,18]. In contrast, others have shown that low oxygen can induce an adipogenic phenotype in telomerase-immortalized human MSCs, though typical adipogenic gene markers were not up-regulated, nor were the lipid morphology characteristic of chemically induced adipocytes *in vitro*[19].

Cytoskeletal organization and oxygen tension have been independently shown to regulate aspects of adipogenic differentiation, but the combined effect has not yet been demonstrated in human adipose-derived stem cells (ASCs). ASCs are an ideal cell type for studying factors that may regulate adipogenesis *in vivo,* as ASCs are found in adult human adipose tissue [20-22], are capable of supporting adipose tissue formation [23], and may participate in adipogenesis of obese adipose tissue [24]. Additionally, ASCs *in vivo* have long protrusions and a branched morphology, not unlike preadipocytes, and in contrast to the spherical and large (diameters up to 100 μm) mature adipocytes [24]. In this study, our objective was to examine how cytoskeletal organization (and apparent tension) and oxygen tension interact to regulate adipogenic differentiation of ASCs *in vitro*. ASCs were adipogenically differentiated in ambient air at 20% O_2_ or in a 5% O_2_ environment to more closely replicate *in vivo* oxygen conditions. To alter cytoskeletal organization and apparent tension of the ASCs, we exposed the cells to the chemical inhibitors cytochalasin D and blebbistatin during the differentiation process. Cytochalasin D reduces cytoskeletal tension by capping the growing ends of f-actin filaments to prevent the addition of monomers, thereby disrupting cytoskeletal organization and reducing tension [25], whereas blebbistatin alters the actin cytoskeleton by inhibiting rigid non-muscle myosin type II crosslinking with actin [26]. To assess the effects of oxygen tension and cytoskeletal inhibition on adipogenesis, we analyzed both early and late markers of adipogenic differentiation, specifically peroxisome proliferator-activated receptor γ (PPARγ), lipoprotein lipase (LPL) and fatty acid binding protein 4 (FABP4) gene expression, as well as adipocyte metabolic function (triglyceride synthesis and accumulation).

## Methods

### Materials

Tissue culture reagents, including Dulbecco’s Modified Eagle Medium (DMEM), fetal bovine serum (FBS), human insulin and penicillin/streptomycin, were purchased from Invitrogen (Carlsbad, CA, USA). Unless otherwise noted, all other chemicals were purchased from Sigma-Aldrich (St. Louis, MO, USA).

### Cell culture

Primary human ASCs used in this study were isolated from subcutaneous adipose tissue samples harvested from the abdomen of three healthy adult female donors (body mass index (BMI) range: 21 to 27; age range: 40 to 59). ASCs were obtained from existing stores and were de-identified and, therefore, were not considered human research subjects and did not require ethics approval; donors provided written informed consent for the collection of the adipose tissue. ASCs were plated at 20,000 cells/cm^2^ in growth medium (DMEM, 10% FBS, and 100 U/mL penicillin and 100 μg/mL streptomycin) and allowed to grow to confluence. Two days post-confluence, growth medium was replaced with adipogenic induction medium containing DMEM, 3% FBS, 33 μM biotin, 17 μM pantothenate, 1 μM insulin, 1 μM dexamethasone, 400 μM 3-isobutyl-1-methylxanthine (IBMX), 5 μM 2,3-thiazolidinedione (TZD), 100 U/mL penicillin and 100 μg/mL streptomycin as previously reported [27]. After one week, the adipogenic induction medium was replaced with maintenance medium consisting of induction medium without IBMX or TZD [27]. Cells were cultured under ambient (20%) or physiological (5%) O_2_ conditions at 37°C in humidified incubators using nitrogen gas to control O_2_ levels. Medium changes were performed every other day for 21 days.

### Cytoskeletal inhibition

To inhibit cytoskeletal tension, cells were treated with either 2 μM cytochalasin D in dimethyl sulfoxide (DMSO) or 50 μM blebbistatin in DMSO at each medium change. The final concentration of DMSO in the medium was 1% v/v and, therefore, 1% DMSO was used in the culture medium as a vehicle control. Concentrations were based on previously published studies [14,15].

### Triglyceride content

One of the hallmarks of adipogenesis is the formation of microscopic lipid vacuoles storing triglycerides. Metabolic analysis of triglyceride content was conducted on the cell lysates as previously described [28]. After 14 and 21 days, cells were trypsinized with 0.05% trypsin-ethylenediaminetetraacetic acid (EDTA) and pelleted by centrifuging at 1,000 × *g* for 10 minutes at 4°C. Cell pellets were washed with phosphate buffered saline (PBS) and re-pelleted. Afterwards, the pellets were dissolved in 50 μL of STES buffer (2 nM NaCl, 50 nM Tris, 10 nM EDTA, 0.001% w/v sodium dodecyl sulfate) and frozen at -20°C until analysis. Previously frozen cells were thawed and sonicated using 60% amplification for one second. Samples were centrifuged at 20,000 × *g* for 10 minutes at 4°C to remove the cellular debris. The supernatant was collected and transferred into a new microcentrifuge tube. Triglyceride content of the supernatant was measured using a triglyceride determination kit, which analyzed the release of glycerol from triglycerides by lipoprotein lipase. Triglyceride values were normalized to total DNA content using Hoechst 33258 dye (Invitrogen).

### Glycerol-3-phosphate dehydrogenase (GPDH; EC 1.1.1.8) enzyme activity

GPDH is a cytoplasmic enzyme involved in the triglyceride biosynthesis pathway converting dihydroxyacetone phosphate into glycerol 3-phosphate [12]. GPDH activity increases during terminal adipogenic differentiation and adipocyte maturation [29]. At days 14 and 21 following adipogenic induction, GPDH activities were measured *in situ* using the method of Sottile and Seuwen [30]. Briefly, cell lysate samples were incubated at 37°C in a solution consisting of 0.1 M triethanolamine, 2.5 mM EDTA, 0.1 mM β-mercaptoethanol, 334 μM nicotinamide adenine dinucleotide (NADH), pH adjusted to 7.7 with 1 M HCl. To start the reaction, dihydroxyacetone phosphate was added to the mix at a final concentration of 0.2 mM. GPDH activity was assessed by measuring the NADH absorbance at 340 nm using a microplate reader (VERSAmax, Molecular Devices, LLC, Sunnyvale, CA, USA). Initial and final absorbance values were recorded after 15 minutes. These values were converted to NADH concentration, which were then used to determine GPDH activity. GPDH activity was normalized to total DNA content determined using Hoechst dye.

### Total DNA content

DNA content of the samples was conducted by staining an aliquot of the cell lysates with Hoechst dye. Fluorescence values for these samples were acquired on a fluorescence microplate reader (SpectraMax Gemini EM, Molecular Devices, LLC, Sunnyvale, CA, USA) using an excitation wavelength of 360 nm and an emission wavelength of 460 nm. These values were then converted to total DNA content by comparing the fluorescence values to a standard curve of fluorescence readings generated from solutions with known DNA concentrations.

### Histology

Samples were rinsed with PBS, fixed for 20 minutes in 4% paraformaldehyde, and washed again with PBS. After fixation, samples were blocked with 3% bovine serum albumin and then stained with Alexa Fluor 488 conjugated phalloidin (Invitrogen) to visualize actin microfilaments. Nuclei were counterstained with Hoechst dye. Images of the phalloidin-stained cells were captured using a Leica DM IL fluorescence microscope (Leica Microsystems, Buffalo Grove, IL, USA) with an excitation wavelength of 495 nm and an emission wavelength of 518 nm. Hoechst images were captured using an excitation wavelength of 350 nm and an emission wavelength of 460 nm.

### Oil Red O staining

Lipid vacuoles were visualized with Oil Red O staining. On days 14 and 21 post-induction, the cells were fixed for 20 minutes using a 4% paraformaldehyde solution. Lipids were stained with a 0.21% (w/v) Oil Red O in 60% (v/v) isopropanol-PBS solution. Samples were then thoroughly washed with PBS over the course of several hours to remove any unbound dye. Images were captured using a Leica DM IL fluorescence microscope using an excitation wavelength of 560 nm and an emission wavelength of 645 nm.

### Quantitative Polymerase Chain Reaction (qPCR)

Each cell sample was homogenized in TRIzol reagent (Invitrogen) and total RNA was extracted according to the manufacturer’s protocol. RNA concentration and quality were assessed spectrophotometrically on the basis of the A_260_/A_280_ readings. Reverse transcription was performed using Superscript III First-Strand Synthesis System (Invitrogen), with 2 μg total RNA reverse transcribed with oligo(dT) primers. qPCR was performed with Brilliant II SYBR Green qPCR Master Mix (Applied Biosystems, Foster City, CA, USA) and the MX3000p qPCR System (Agilent Technologies, Santa Clara, CA, USA). Primer pairs were designed and optimized for qPCR analysis of expression levels of 18S rRNA (forward 5′-*gactcaacacgggaaacctcacc*-3′; reverse 5′-*accagacaaatcgctccaccaact*-3′), PPARγ (forward 5′-*aatgccatcaggtttgggcgga*-3′; reverse 5′-*cgccctcgcctttgctttgg*-3′), LPL (forward 5′-*gctcgtgctgactctggccg*-3′; reverse 5′-*tcttctttggtcggcggcgg*-3′), and FABP4 (forward 5′-*tgataaactggtggtggaatgcgtc*-3′; reverse 5′- *ctctctcataaactctcgtggaagtg*-3′). Expression levels relative to 18S rRNA were calculated using the delta-delta cycle threshold method.

### Statistical analysis

All experiments were performed in triplicate with cells from three donors. Comparisons of treatment group means as a function of oxygen tension or time were performed using unpaired Student’s *t*-tests. Comparisons of treatment group means as a function of cytoskeletal tension inhibitor were conducted using one-way ANOVA with Tukey’s *post-hoc* test. Treatment group means were deemed to be statistically significant when *P <*0.05.

## Results

### Cytoskeletal organization

**Figure 1:**
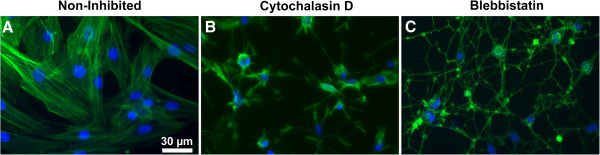
**Cytoskeletal morphology as a function of inhibitor treatment.** Alexa Fluor 488 conjugated phalloidin (green) staining showed the actin microfilament structure of non-inhibited adipose-derived stem cells (ASCs) **(A)**, and the disrupted cytoskeleton of ASCs cultured with 2 μM cytochalasin D **(B)** and 50 μM blebbistatin **(C)**. ASC nuclei were counterstained with Hoechst (blue). Cytochalasin D inhibited actin polymerization and blebbistatin inhibited non-muscle myosin type II activity, altering cell morphology. Scale bar = 30 μm.

Control ASCs showed spread actin microfilament formation (Figure 1A). Cells treated with cytochalasin D developed a shrunken morphology as the result of actin microfilament degradation, evident by the presence of altered and shorter actin microfilaments as compared to the control group (Figure 1B). ASCs treated with blebbistatin were incapable of maintaining a spread cell morphology, presumably because crosslinking between non-muscle myosin II and actin filaments was inhibited. Phalloidin-stained images showed that the actin microfilaments in cells of the blebbistatin group were shorter and the network was disrupted as compared to the control group (Figure 1C).

### Proliferation

**Figure 2:**
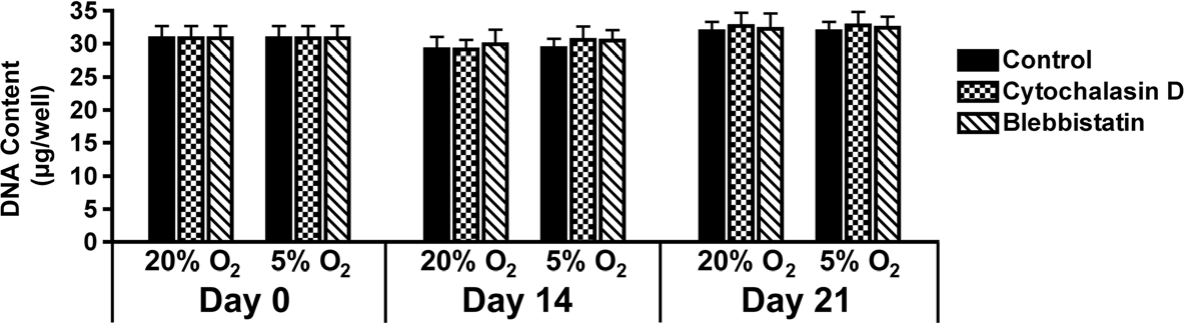
**Proliferation as a function of oxygen levels and cytoskeletal inhibition.** To assess proliferation, DNA content (μg/well) was analyzed after 0, 14 and 21 days of differentiation. Adipose-derived stem cells (ASCs) were exposed to 20% O_2_ and 5% O_2_ conditions, and treated with cytochalasin D (checkered), blebbistatin (striped) or vehicle control (solid). Oxygen levels and cytoskeletal conditions did not significantly change DNA content over 21 days.

Total DNA content at different time points was assessed to examine whether reduced oxygen level or altered cytoskeletal organization compromised viability or affected proliferation (Figure 2). DNA content did not significantly change (*P* >0.05) as a function of oxygen tension, cytoskeletal inhibition, or time over the course of the 21-day differentiation period.

### Adipogenic gene expression

**Figure 3:**
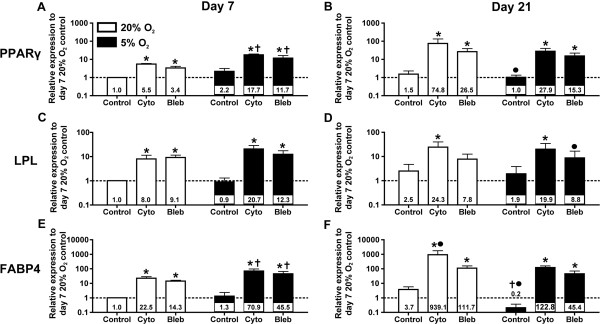
**Adipogenic gene expression as a function of oxygen levels and cytoskeletal inhibition.** Adipose-derived stem cells (ASCs) were cultured under 20% O_2_ (white) and 5% O_2_ (black) conditions, and treated with cytochalasin D (cyto), blebbistatin (bleb) or vehicle control (control). All transcript levels were normalized to 18 S ribosomal RNA, and then to 20% O_2_, non-inhibited (control) samples on Day 7. Peroxisome proliferator-activated receptor γ (PPARγ) **(A, B)**, lipoprotein lipase (LPL) **(C, D)** and fatty acid binding protein 4 (FABP4) **(E, F)** expression were analyzed on days 7 and 21. Culturing under 5% O_2_ conditions significantly decreased FABP4 in the non-inhibited control group compared to the 20% O_2_ control group at Day 21. Cytoskeletal inhibitors significantly increased adipogenic gene expression regardless of oxygen levels or time point as compared to non-inhibited controls. Cytoskeletal inhibitors applied under 5% O_2_ conditions led to a significant increase in PPARγ **(A)** and FABP4 **(E)** gene expression as compared to the corresponding 20% O_2_ inhibited samples at Day 7. Values are reported as mean ± SEM of n = 3 donors; * = significant change relative to non-inhibited control under the same oxygen condition with *P* <0.05; † = significant change relative to corresponding 20% O_2_ sample with *P* <0.05; • = significant change relative to corresponding Day 7 sample with *P* <0.05. Significance was only reported for trends that were consistent for all three cell donors.

The effects of oxygen tension and cytoskeletal inhibitors on adipogenesis were assessed based on gene expression levels for PPARγ, LPL and FABP4 (Figure 3). In the absence of a cytoskeletal inhibitor, LPL expression of cells under 5% O_2_ decreased slightly at Day 7 as compared to control cells under 20% O_2_, but was not significantly different (*P* >0.05). At Day 7 under 5% O_2_, there was a numerical increase in PPARγ and FABP4 expression levels, but these trends were inconsistent across the three donors and were not statistically significant. At Day 21, FABP4 expression decreased significantly (18.5-fold less; *P* <0.05) under 5% O_2_ as compared to 20% O_2_ (Figure 3F). Expression of PPARγ and LPL also decreased (1.5- and 1.3-fold less, respectively), but these differences were not significant. From Day 7 to Day 21, PPARγ and FABP4 gene expression levels slightly increased under 20% O_2_ (1.5- and 3.7-fold; *P* >0.05), but PPARγ and FABP4 significantly decreased under 5% O_2_ (2.2- and 6.5-fold; *P* <0.05; Figure 3). LPL increased under 20% O_2_ (2.5-fold) and 5% O_2_ (2.1-fold) from days 7 to 21; however, neither up-regulation was significant (*P* >0.05).

Under 20% O_2_, the addition of cytochalasin D and blebbistatin to the differentiation medium significantly increased the expression levels of all three adipogenic markers at Day 7, as compared to non-inhibited controls (*P* <0.05; Figure 3). Cytochalasin D and blebbistatin significantly increased the expression of PPARγ by 5.5- and 3.4-fold (Figure 3A), LPL by 8.0- and 9.1-fold (Figure 3C), and FABP4 by 22.5- and 14.3-fold (Figure 3E), respectively, as compared to non-inhibited controls (*P* <0.05). At 21 days, cytochalasin D and blebbistatin treatment significantly increased PPARγ expression by 49.9- and 17.7-fold (Figure 3B), LPL expression by 9.7-fold (cytochalasin D only; Figure 3D) and FABP4 expression by 253.8- and 30.2-fold (Figure 3F), respectively, as compared to non-inhibited controls (*P* <0.05).

Combining inhibitor treatment under reduced oxygen tension led to similar increases in PPARγ, LPL and FABP4 expression levels at Day 7 as compared to inhibitor treatment under ambient oxygen tension (Figure 3). However, the combined effects were substantially greater in magnitude. Under 5% O_2_, treatment with cytochalasin D and blebbistatin significantly increased the expression levels of PPARγ by 8.0- and 5.3-fold (Figure 3A), LPL by 23.0- and 13.7-fold (Figure 3C), and FABP4 by 54.5- and 35.0-fold (Figure 3E), respectively, as compared to the corresponding control cells under the same oxygen tension at Day 7 (*P* <0.05). Expression of PPARγ under 5% O_2_ with cytochalasin D and blebbistatin treatment did not change significantly from Day 7 to Day 21 (*P* >0.05). Blebbistatin treatment at 21 days under 5% O_2_ resulted in a significant decrease in LPL expression, but no change was seen with cytochalasin D treatment, compared to the corresponding 5% O_2_, Day 7 sample. Expression of FABP4 under 5% O_2_ also remained constant in blebbistatin-treated cells from Day 7 to Day 21, but increased in cytochalasin D treated cells, though not significantly. Similar to ambient oxygen tension, inhibitor treatment led to significant increases in all gene expression levels under reduced oxygen tension at Day 21 as compared to non-inhibited control groups with the exception of LPL expression in the blebbistatin treated group. Under 5% O_2_, treatment with cytochalasin D and blebbistatin significantly increased the expression levels of PPARγ by 27.9- and 15.3-fold (Figure 3B), LPL by 10.5-fold (cytochalasin D only; Figure 3D), and FABP4 by 614.0- and 227.0-fold (Figure 3F), respectively, as compared to the control cells under reduced oxygen tension at Day 21 (*P* <0.05).

### Lipid metabolism

**Figure 4:**
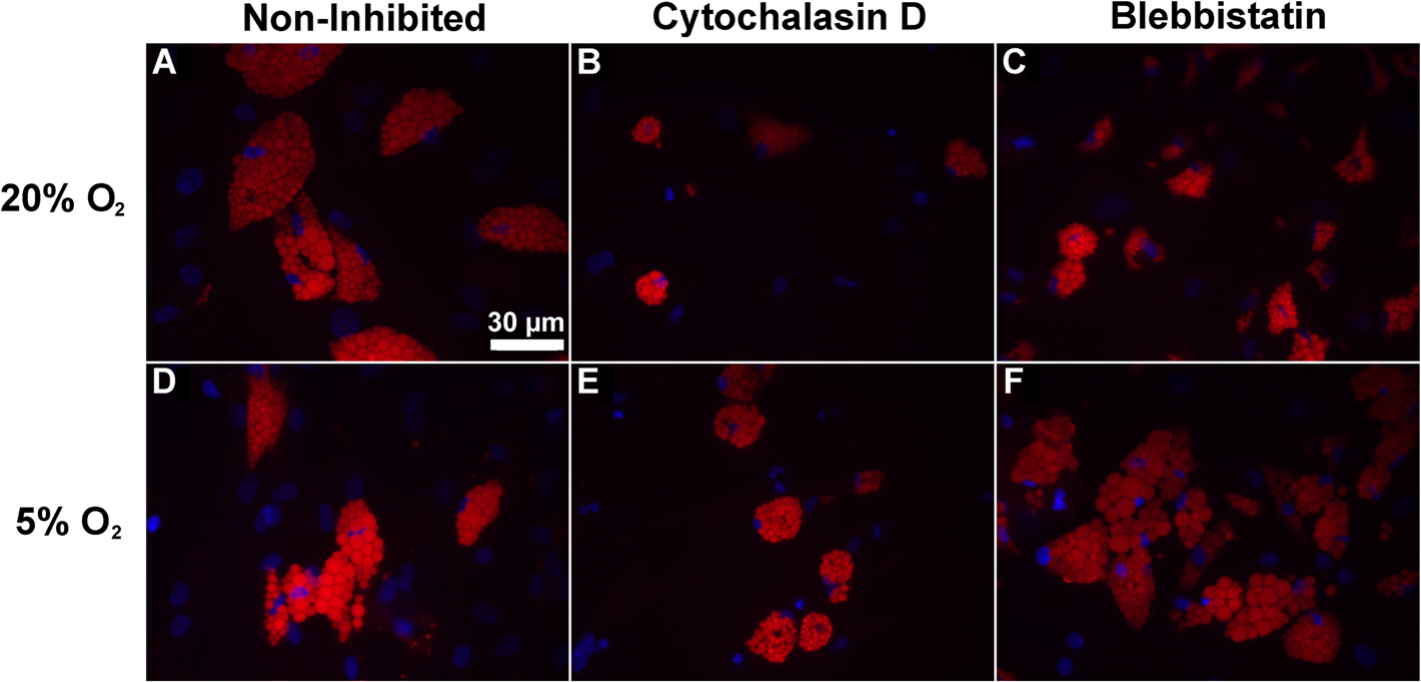
**Lipid morphology as a function of oxygen levels and cytoskeletal inhibition.** Oil Red O staining (red) qualitatively demonstrated changes in lipid accumulation in adipose-derived stem cells (ASCs) that had been differentiated for 21 days in 20% O_2_ and 5% O_2_ conditions. ASCs under 20% O_2_ conditions without cytoskeleton inhibitor treatment **(A)** appeared to have a higher percentage of Oil Red O positive cells compared to ASCs under 5% O_2_ conditions without inhibitor treatment **(D)**. The addition of either cytoskeletal inhibitor, cytochalasin D **(B,E)** or blebbistatin **(C,F)**, appeared to increase the percentage of lipid stained cells regardless of oxygen conditions compared to non-inhibited ASCs **(A,D)**. ASC nuclei were counterstained with Hoechst (blue). Scale bar = 30 μm.

**Figure 5:**
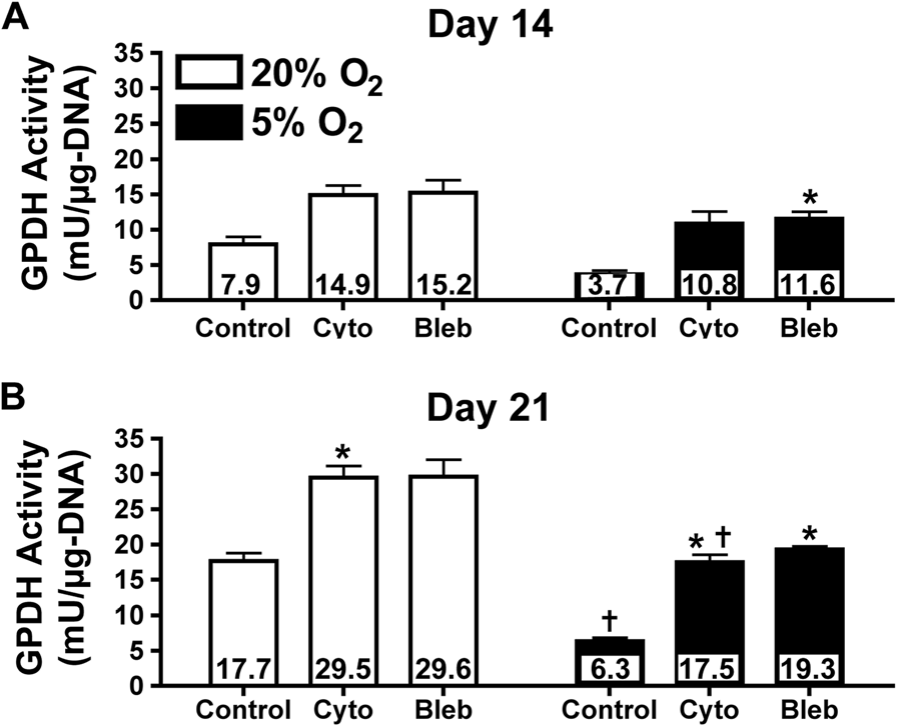
**Glycerol-3-phosphate dehydrogenase (GPDH) activity as a function of oxygen levels and cytoskeletal inhibition.** GPDH activity was analyzed after 14 and 21 days of differentiation. Adipose-derived stem cells (ASCs) were cultured under 20% O_2_ (white) and 5% O_2_ (black) conditions, and treated with cytochalasin D (cyto), blebbistatin (bleb) or vehicle control (control). In control groups, 5% O_2_, reduced GPDH activity after 14 **(A)** and significantly at 21 **(B)** days, compared to 20% O_2_. Conversely, cytochalasin D and blebbistatin increased GPDH activity compared to non-inhibited samples, regardless of oxygen condition after 14 (A) and 21 (B) days. Values were reported as mean ± SEM with n = 3 cell donors; * = significant change relative to non-inhibited control under the same oxygen condition with *P* <0.05; † = significant change relative to corresponding 20% O_2_ sample with *P* <0.05. Significance was only reported for trends that were consistent for all three cell donors.

**Figure 6:**
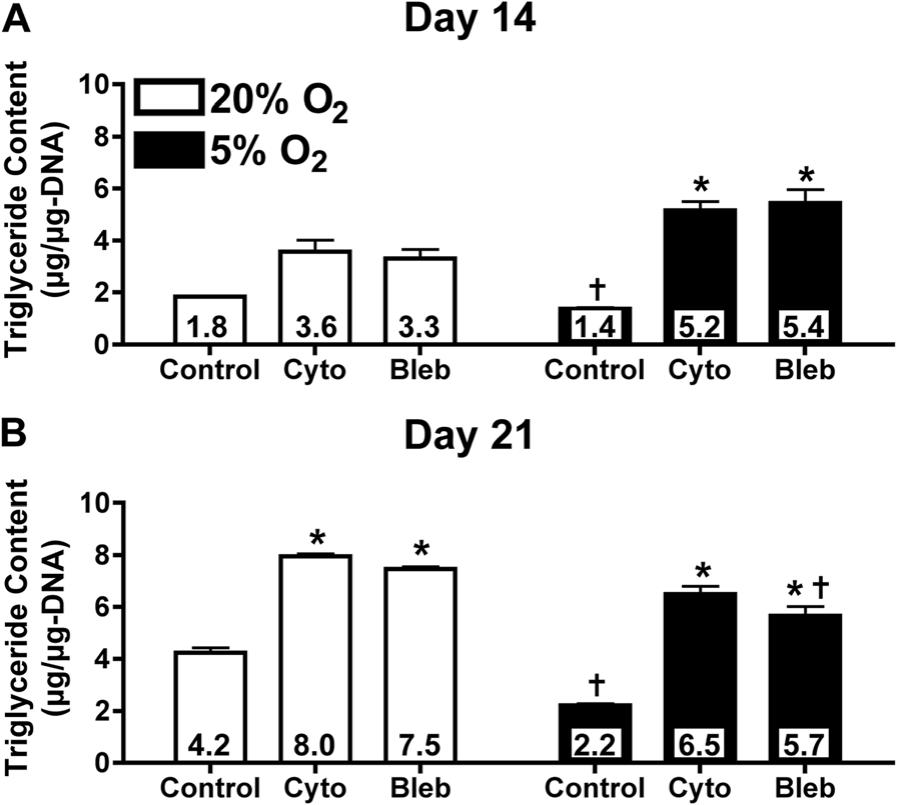
**Triglyceride content as a function of oxygen levels and cytoskeletal inhibition.** Triglyceride content was analyzed after 14 and 21 days of differentiation. Adipose-derived stem cells (ASCs) were cultured under 20% O_2_ (white) and 5% O_2_ (black) conditions, and treated with cytochalasin D (cyto), blebbistatin (bleb) or vehicle control (control). In control groups, 5% O_2_ significantly reduced triglyceride content compared to 20% O_2_ after 14 **(A)** and 21 **(B)** days. Conversely, cytochalasin D and blebbistatin increased triglyceride content compared to non-inhibited samples, regardless of oxygen condition after 14 (A) and 21 (B) days. Values were reported as mean ± SEM with n = 3 cell donors; * = significant change relative to non-inhibited control under the same oxygen condition with *P* <0.05; † = significant change relative to corresponding 20% O_2_ sample with *P* <0.05. Significance was only reported for trends that were consistent for all three cell donors.

As a functional measure of the effect of the oxygen tension and cytoskeletal tension on adipogenic differentiation, we analyzed lipid metabolism by assessing lipid droplet formation, cytoplasmic GPDH activity and triglyceride content. Regardless of oxygen tension or cytoskeletal inhibitor treatment, all experimental groups showed microscopic lipid droplets after 21 days following adipogenic induction as indicated by Oil Red O staining (Figure 4). In control cells without inhibitor treatment, reducing the oxygen tension to 5% O_2_ lowered both GPDH activity (Figure 5) and triglyceride accumulation (Figure 6). At 14 days, triglyceride content was significantly reduced by 1.3-fold (*P* <0.05) with 5% O_2_ (Figure 6A). At 21 days, GPDH activity (Figure 5B) and triglyceride content (Figure 6B) were significantly reduced by 2.8- and 1.9-fold, respectively (*P* <0.05). Adding the cytoskeletal inhibitors increased GPDH activity and triglyceride accumulation under both ambient and reduced oxygen tensions, compared to non-inhibited controls. The up-regulation in GPDH activity under reduced oxygen tension was more pronounced after 21 days, when the effects of both cytochalasin D and blebbistatin were significant (*P* <0.05; Figure 5B). Similar trends with cytochalasin D and blebbistatin treatment were observed for triglyceride accumulation, although triglyceride content at 21 days was only slightly larger than at 14 days (Figure 6). After 21 days under 5% O_2_, cytochalasin D and blebbistatin treatment increased GPDH activity of cells (17.5 and 19.3 mU/μg-DNA, respectively) to GPDH activity levels that were similar to non-inhibited control cells under 20% O_2_ (17.7 mU/μg-DNA) (Figure 5B). Under 5% O_2_, cytochalasin D and blebbistatin treatment elevated triglyceride content compared to non-inhibited control cells under 20% after both 14 and 21 days (Figure 6).

## Discussion

To better understand the role of mechanical and environmental factors in adipogenesis, we have examined how altered cell morphology (and the associated decrease in cytoskeletal tension) may synergistically interact with physiological oxygen conditions to regulate adipogenic differentiation of ASCs. In this study, we demonstrated a significant influence of cytoskeletal organization on adipogenic differentiation under both physiological and ambient oxygen conditions. Treatment with cytoskeletal inhibitors to reduce apparent cytoskeletal tension in primary adult human ASCs enhanced the expression of adipogenic marker genes as well as functional indicators of lipid metabolism.

Combining physiological oxygen and cytoskeletal inhibition had a dramatic impact on ASC adipogenesis. Under physiological oxygen tension with cytoskeletal inhibition, PPARγ and FABP4 gene expression levels were significantly increased following seven days of induction compared to ambient oxygen and non-inhibited controls (Figure 3). These trends were maintained following 21 days of induction. Exposure to physiological oxygen tension for 21 days significantly down-regulated FABP4 gene expression, but cytoskeletal inhibition reversed this decrease (Figure 3). Physiological oxygen tension also significantly decreased GPDH activity (Figure 5) and triglyceride accumulation (Figure 6). These decreases in functional markers of mature adipocytes may be related to the diminished FABP4 gene expression.

Based on our results, we postulate that within the *in vivo* ASC niche at physiological oxygen levels, cytoskeletal tension may regulate adipose tissue expansion. The combined effect of cytoskeletal tension and physiological oxygen levels on ASCs has not been previously reported. Our observations on the effects of either cytoskeletal inhibition or reduced oxygen tension, applied individually, are qualitatively consistent with previously published studies using other types of stem cells. Reducing cytoskeletal tension via cytochalasin D and blebbistatin induced rounded cell morphology and up-regulated adipogenesis in MSCs [14] and C3H10T1/2 cells, an immortalized MSC line [15]. *In vivo* and *in vitro*, preadipocytes possess a fibroblastic morphology; however, mature adipocytes are more spherical [8,11]. The mechanisms driving the morphological change during adipogenesis *in vivo* remain unclear*.* In the present study, we chose to induce these changes *in vitro* through the use of chemical inhibitors that target intracellular cytoskeletal tension. While cytochalasin D and blebbistatin act through different mechanisms to disrupt the actin cytoskeleton, they produced similar cell shape changes and enhanced adipogenesis, suggesting that the enhancement is due to actin disassembly and reorganization.

Physiological oxygen tension, which is substantially lower than the ambient oxygen tension typically used in cell culture studies, has been shown to inhibit stem cell differentiation and aid in maintaining pluripotency through hypoxia-inducible factor (HIF)-regulated pathways [31]. Previous *in vitro* adipogenesis studies using low oxygen conditions or chemical treatments mimicking hypoxia have demonstrated that low oxygen ten-sion inhibits or reduces adipogenic differentiation in preadipocyte cell lines [6,17,18]. Studies with primary stem cells found that low oxygen conditions not only reduced lineage commitment in ASCs [32], but extended the differentiation potential in adipose tissue stromal cells [33] and bone marrow-derived MSCs [34] compared to ambient oxygen conditions. Additionally, when cultured under low oxygen, human MSCs showed diminished adipogenic differentiation and FABP4 gene expression [35]. Similar to the MSCs, we demonstrated suppression of adipogenic differentiation marker expression (FABP4, GPDH and triglycerides) in the ASCs under physiological oxygen conditions. In contrast, ASCs cultured under chondrogenic conditions and 5% O_2_ significantly increased cartilaginous matrix production while cell proliferation decreased [22]. Taken together, these and our findings demonstrate that reducing the oxygen tension to a physiological level can differentially regulate the switch between growth and differentiation in ASCs.

The FABP4 gene encodes an intracellular fatty acid binding protein that is found in differentiated adipocytes [36]. During adipogenesis, the adipogenic transcription factors PPARγ and CCAAT/enhancer binding protein α (C/EBPα) induce FABP4 mRNA expression, resulting in increased FABP4 protein synthesis [37]. Studies with FABP4-deficient mice revealed that FABP4 plays a role in adipocyte fatty acid metabolism, triglyceride storage and insulin resistance [38]. Our results showed that low oxygen tension reduced FABP4 gene expression relative to ambient oxygen tension in ASCs at 21 days (Figure 3). This was consistent with an earlier study involving MSCs [35], though the exact mechanism for FABP4 reduction was not examined. We did not find a corresponding decrease in PPARγ, which typically regulates FABP4 expression, suggesting that FABP4 expression may also be controlled through a different mechanism. The ASCs with reduced FABP4 expression also had significantly decreased triglyceride content and GPDH activity, suggesting that lipid synthesis and storage can also respond to lowered oxygen tension via a PPARγ-independent mechanism. Interestingly, cytoskeletal inhibition abolished the physiological oxygen-mediated down-regulation of adipogenic markers, suggesting that cytoskeletal tension may act as an overriding regulator of ASC-to-adipocyte differentiation *in vivo*.

Our results indicate there may be significant crosstalk between the oxygen tension and cytoskeletal pathways to regulate adipogenesis, though specific signaling pathways have yet to be elucidated. Unfortunately, current literature does not provide a clear picture of how oxygen tension and cytoskeletal tension pathways interact. Several pathways regulate adipogenesis via cytoskeletal tension, including the RhoA/Rho-associated protein kinase (ROCK) pathway. ROCK is capable of phosphorylating several molecules, including myosin light chain and myosin light chain phosphatase. The phosphorylation of these molecules increases non-muscle myosin type II activity, which regulates the tension between actin microfilaments. Several studies have shown that RhoA/ROCK-mediated cytoskeletal tension can be a key regulator of adipogenesis in stem cells [14,15]. Treatment of the cells with cytochalasin D or blebbistatin led to an up-regulation of adipogenic gene expression (Figure 3) and an increase in GPDH activity (Figure 5) and triglyceride content (Figure 6) suggesting that the RhoA/ROCK pathway could be involved. However, the previous studies on cytoskeletal tension and adipogenesis were conducted under ambient oxygen conditions and did not address the effects that low oxygen tension would have on the differentiation process. The few studies that did examine how the RhoA/ROCK pathway functions under low oxygen suggest that RhoA activation induces HIF-1α expression [39]. HIF-1α is regarded as the primary transcription factor of hypoxia-regulated functions and has been linked to the down-regulation of adipogenesis *in vitro* by inhibiting the transcription of C/EBPβ and PPARγ in 3T3-L1 preadipocytes [6,18]. However, the studies linking HIF-1α and RhoA did not indicate whether downstream components of the RhoA/ROCK pathway, specifically, cytoskeletal tension, were involved in regulating HIF-1α [39].

A potential limitation of this study is the use of small molecule chemical inhibitors to disassemble the actin microfilaments (via cytochalasin D) or to inhibit non-muscle myosin type II binding to actin (via blebbistatin) to reduce cytoskeletal tension and alter cell shape. These inhibitors affect the cell morphology leading to cell rounding, whereas cellular shape may also be regulated by extracellular matrix factors, such as substrate stiffness. Exploring external mechanical factors with an intact actin cytoskeleton may provide additional insights into how cell shape influences and is controlled during adipogenesis *in vivo.* ASCs and mature adipocytes occupy unique extracellular niches within the adipose tissue environment [24], and differences between niche mechanical properties could have a significant impact on actin cytoskeleton assembly, morphology and mechanotransduction. For instance, ASCs cultured on a soft substrate demonstrated an amorphous actin cytoskeleton and rounded morphology, compared to the pronounced actin filaments and fibroblastic morphology associated with stiff substrates [40]. Our current study lays the foundation for investigating synergetic effects of the cytoskeleton and oxygen tension. Future studies will focus on regulating the actin cytoskeleton through other means such as matrix stiffness, which has previously been shown to influence adipogenesis [7]. A second potential limitation of this study and nearly all *in vitro* adipogenic differentiation studies is the use of synthetic chemical cocktails to induce differentiation. This methodology is well established to study stem cell biology, but is likely not entirely representative of *in vivo* differentiation conditions. Therefore, we must use caution when extrapolating *in vitro* results to *in vivo* differentiation. Additionally, this study was conducted in a typical two-dimensional monolayer culture, making the results easier to compare with prior studies. Future studies are warranted to conduct experiments in three-dimensional culture systems that more closely mimic the *in vivo* microenvironment.

## Conclusion

In summary, we have found that altering cytoskeletal organization and associated tension in ASCs enhances adipogenic differentiation. Physiological oxygen levels depressed adipogenic differentiation markers, but this down-regulation was abolished with cytoskeletal inhibition. This study demonstrates the profound and perhaps overriding influence that the cytoskeleton has in regulating the fate decisions of ASCs. Further studies are still needed to elucidate the molecular pathways. The outcomes of these studies could have implications in several areas, including obesity research as well as developing *in vitro* adipose tissue models.

## Abbreviations

ASC: Adipose-derived stem cell; BMI: Body mass index; C/EBP: CCAAT/enhancer binding protein; DMEM: Dulbecco’s Modified Eagle Medium; DMSO: Dimethyl sulfoxide; EDTA: Ethylenediaminetetraacetic acid; FABP4: Fatty acid binding protein 4; FBS: Fetal bovine serum; GPDH: Glycerol-3-phosphate dehydrogenase; HIF: Hypoxia-inducible factor; IBMX: 3-isobutyl-1-methylxanthine; LPL: Lipoprotein lipase; MSC: Mesenchymal stem cell; NADH: Nicotinamide adenine dinucleotide; PBS: Phosphate buffered saline; PPARγ: Peroxisome proliferator-activated receptor gamma; qPCR: Quantitative polymerase chain reaction; ROCK: Rho-associated protein kinase; SEM: Standard error of mean; TZD: 2,3-thiazolidinedione.

## Competing interests

The authors declare they have no competing interests.

## Authors’ contributions

This study was conceived, designed and coordinated by CKK, KL and ZAS. Cell culture and PCR assays were conducted by ZAS. Functional marker assays were carried out by ZAS with assistance by JKS. Statistical analysis of the results was performed by ZAS. The manuscript was written and revised by CKK, JKS, KL, NRS and ZAS. All authors read and approved the final manuscript.
